# Laboratory Scale Continuous Flow Systems for the Enantioselective Phase Transfer Catalytic Synthesis of Quaternary Amino Acids

**DOI:** 10.3390/molecules28031002

**Published:** 2023-01-19

**Authors:** Milena Krstić, Sergio Rossi, Miguel Sanz, Alessandra Puglisi

**Affiliations:** 1 Dipartimento di Chimica, Università degli Studi di Milano, Via Golgi 19, 20133 Milano, Italy; 2 Taros Chemicals, GmbH & Co. KG Emil-Figge-Str 76a, 44227 Dortmund, Germany

**Keywords:** continuous flow, quaternary stereocenter, phase transfer catalysis (PTC), enantioselective synthesis, packed-bed reactor, continuous stirred tank reactor, asymmetric catalysis

## Abstract

The use of stereoselective phase-transfer catalysis as a reliable method for the enantioselective synthesis of optically active α-amino acid derivatives using achiral Schiff base esters has been well-developed in batch in the last 40 years. Recently, continuous flow technology has become of great interest in the academy and industry, since it offers safer process operating conditions and higher efficiency compared to a traditional batch processing. Herein, we wish to report the first example of enantioselective phase transfer benzylation of alanine Schiff base ester, under continuous flow conditions. Two different methodologies were investigated: a liquid-solid phase transfer catalytic benzylation using a packed-bed reactor and a liquid-liquid phase transfer catalytic benzylation in continuous stirred-tank reactors. Liquid-liquid phase transfer process in flow showed slightly better productivity than the batch process, while solid-liquid phase transfer benzylation proved much more advantageous in terms of productivity and space-time yield. Furthermore, continuous flow system allowed the isolation of benzylated product without any work up, with a significant simplification of the process. In both cases, phase transfer asymmetric benzylation promoted by Maruoka catalyst demonstrated high enantioselectivity of target quaternary amino ester in flow, up to 93% ee.

## 1. Introduction

The asymmetric synthesis of α,α-disubstituted amino acids has been extensively explored in the last four decades. Due to the biochemical and medicinal importance of amino acids, the reproducible synthesis of non-proteinogenic amino acids as a research area has gained a lot of attention in the past period [1,2]. Unnatural α,α-dialkyl amino acids play a specific role in the design of peptides [3,4]. They can be potent enzyme inhibitors and significant fragments for the synthesis of different biologically active compounds. Conformationally constrained and stereochemically stable due to the presence of quaternary carbon atoms, unusual α,α-disubstituted amino acids may serve as useful tool in protein research. Inclusion of rigid amino acids into peptides can enhance their activity, bioavailability, and binding selectivity [1,5], but they represent relevant components also of pharmaceuticals, agrochemicals, food additives and various natural products [6,7]. 

Phase-transfer catalysis in batch has been recognized as the most established synthetic method for the enantioselective synthesis of optically active α-amino acid derivatives using Schiff base esters [8,9]. This strategy was applied in the synthesis of nonproteinogenic amino acids bearing stable α-quaternary carbon center, a significant building block playing a vital role in biological systems [10,11,12]. Quaternary ammonium salts derived from the Cinchona alkaloids, such as O’Donnell, Corey, Lygo, and spiro-derivatives like Maruoka type catalysts, were mainly used as phase-transfer catalysts to perform asymmetric alkylation/benzylation of amino acid derivatives [8,9,12,13,14,15,16,17]. 

However, scaling up of phase transfer catalytic reactions in batch can be a challenging process due to various factors: reactor size and shape, stirring rate, which directly affects the heat and mass transfer performances, influencing the overall reaction rate and temperature distribution in batch reactor [18]. Phase transfer catalysis is a complex system in which the catalyst constantly moves from one phase to another, and therefore it makes the measurement of mass transfer problematic [19]. Microreactors/packed-bed reactors and continuous flow technologies have shown certain advantages compared to batch conditions in phase transfer catalysis, in terms of better control over the process, faster mass and heat transfer, and shorter reaction times [19,20,21,22,23,24,25]. The challenge in phase transfer catalysis is the control of drop size distribution between the two phases, the size of the interfacial surface area, the rate of catalyst transfer, and selectivity [19]. Thus, for the development of efficient phase transfer catalysis in flow, many parameters are important, such as reactor design, aqueous and organic flow rates, the slug length, the diameter of the channels, residence time [19].

So far, many phase transfer reactions were described in continuous flow mode, but almost no examples are known of stereoselective catalytic in-flow transformations. For example, in 2003 Kobayashi et al. illustrated very efficient phase transfer alkylation of β-keto esters in a microchip reactor using tetrabutylammonium bromide [20]. In 2006, Okamoto studied the influence of microchannel flow process on the yield in alkylation of malonic ester with a phase transfer catalyst (tetrabutylammonium hydrogen sulfate) [21]. In 2012, De Zani and Colombo reported an efficient continuous flow conditions for the liquid-liquid phase transfer *O*-alkylation of substituted phenols with alkyl halides, by using T-junction inlet before the reactor, to form the segmented flow. The target phenyl ethers were obtained in very good yields, in very short reaction times [24]. Afterwards, Reichart, Kappe and Glasnov investigated continuous-flow technology for the biphasic phase transfer *O*- and *S*-alkylation of phenols and thiophenols [25]. They tested different flow set up: a stainless-steel coil reactor, a packed-bed reactor (filled with stainless steel beads) and a glass chip microreactor. All of them showed excellent performances, showing more than 93% conversion [25]. But there are scarce data in the literature about phase transfer catalysis in flow for the synthesis of chiral amino acid derivatives. The only reported example on this topic was done by Jensen and co-workers. They have conducted liquid-liquid asymmetric benzylation of *N*-(diphenylmethylene) glycine tert-butyl ester by Cinchonidine-derived phase transfer catalyst in continuous stirred-tank reactor (CSTR), using various residence times and agitation intensities, to form tertiary amino acid derivatives [26]. 

To the best of our knowledge, there is no published study for the asymmetric phase transfer preparation of amino acid derivatives containing a quaternary stereocenter under continuous flow regime. Herein, we report two different in continuo methodologies to generate quaternary amino acids: (1) liquid-liquid phase transfer catalysis using continuous-stirred tank reactor (CSTR); (2) solid-liquid phase transfer catalysis using a packed-bed reactor.

## 2. Results

### 2.1. Preliminary Tests in Batch 

**Figure 1:**
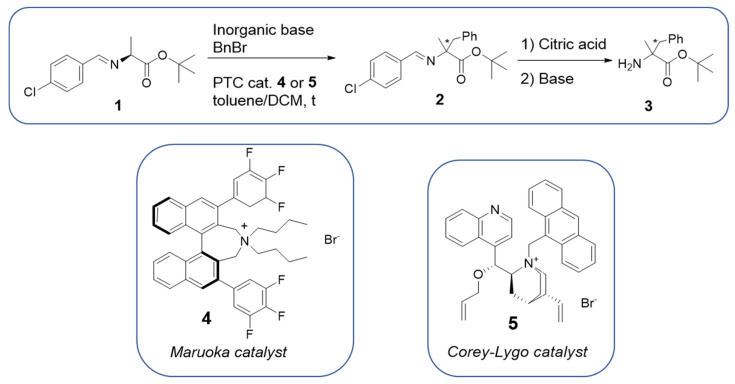
Phase transfer model reaction for continuous flow methodology.

As already mentioned, asymmetric phase transfer alkylation/benzylation/allylation of Schiff base esters in batch, to generate tertiary or quaternary amino acid derivatives, is very well described in the literature [8,9,12,13,14,15,16,17]. Nevertheless, batch experiments have been conducted in order to make representative benchmarking model relative to continuous flow experiments in terms of productivity. Asymmetric phase transfer benzylation of L-alanine imine **1**, for the formation of quaternary stereocenter was used as a model reaction in batch as well as in continuous flow, Figure 1. Standard batch reaction conditions are listed in Table 1 (liquid-liquid or solid liquid phase transfer catalysis). Due to the low stability of compound **2**, hydrolysis of benzylated imine with citric acid followed by basification (K_2_CO_3_, NaHCO_3_ or NaOH), led to the generation of amino ester **3**, stable compound for determination of enantioselectivity. Preliminary experiments conducted on the benzophenone imine of L-alanine tert-butyl ester, a more stable ketimine, easy to isolate in high yields, showed a lower reactivity compared to imine **1**. For this reason, we decided to continue our investigations on compound **1**.

As shown in Table 1, the solid/liquid methodology outperformed the liquid/liquid one, affording yield up to 67% in shorter reaction time (entry 1 vs. entries 2–5). The chiral catalyst did not affect the yield of product **3**: both catalyst **4** and **5** afforded the product in satisfactory yields. However, the best results in terms of enantioselectivity of amino ester **3** was achieved with Maruoka catalyst **4** (up to 90% ee) [8,9,12,13,14,15,16,17].

### 2.2. Liquid-Liquid Enantioselective Phase Transfer Benzylation of L-alanine Imine in Flow 

While the enantioselective phase transfer benzylation of imine of glycine in a CSTR is documented in a paper by Jensen and co-workers, [26] the enantioselective phase transfer catalytic alkylation of more sterically hindered α-amino acids under continuous flow conditions is unprecedent in the literature. Asymmetric phase transfer benzylation of the Schiff base of L-alanine and 4-chlorobenzaldehyde **1**, in continuous flow for the formation of quaternary amino acid derivative **2** was used as a model reaction for our investigations (Figure 1). The work by Jensen [26] was used as a reference flow system for our studies and it showed perfect reproducibility (see Supplementary Materials for further details).

In the case of liquid-liquid phase transfer benzylation of L-alanine imine, all experiments were conducted in continuous stirred tank reactor (CSTR). Miniature continuously stirred tank reactors (CSTRs) are agitated vessels with inlets and outlets providing intensive agitation inside the sealed miniaturized chamber [26,27]. They are very convenient for multiphasic reactions, but also designed to handle reactions that involve solid precipitates [26]. CSTR modules are made of heat and chemical resistant materials, such as high-performance engineering plastic PEEK (polyether ether ketone) and borosilicate glass cover. To ensure efficient stirring inside the CSTR units, special PTFE cross stirrer bars are designed. Standard PTFE tubing (1/8″ OD (outer diameter), 1/16 ID (inner diameter)) are used to connect CSTR modules.

**Figure 2:**
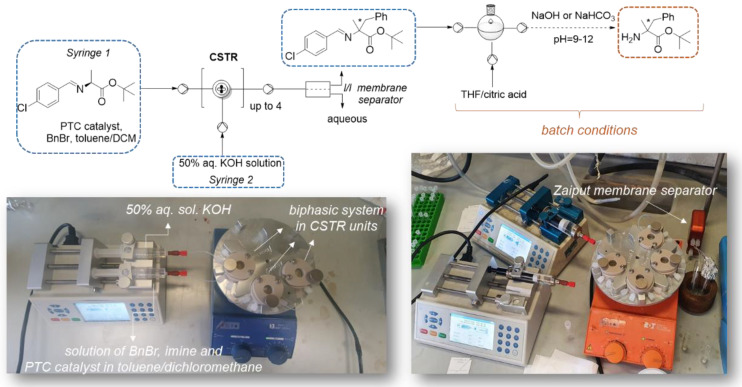
Liquid-liquid phase transfer benzylation in CSTR.

Our experimental setup is illustrated in Figure 2: one syringe was filled with the liquid base (50% aqueous solution of KOH), while the second syringe was filled with the solution of L-alanine imine **1**, benzyl bromide and phase transfer catalyst (Maruoka **4** or Corey-Lygo **5** catalyst) in toluene/dichloromethane. The two solutions (aqueous and organic) were delivered by a syringe pump to a series of CSTRs (up to 4 units) kept at ambient temperature and under agitation ~1100 RPM. At the end of the reaction time, the two phases were separated by a membrane separator (Zaiput separation device). After the separation, the organic solvents were removed, and the residue was analyzed by ^1^H-NMR. The deprotection of benzylated imine **2** to get quaternary amino ester **3** was conducted under batch conditions with citric acid in THF, followed by treatment with inorganic base (NaOH or NaHCO_3_). 

In Table 2 we report selected results, obtained by exploring different reaction conditions: phase transfer catalyst, equivalents of benzyl bromide, residence time, flow rate of organic phase (op) or water phase (wp), ratio of solvents, number of CSTR units. We underline here that toluene is the solvent of choice that guarantees high enantioselectivity for this reaction [see [4] and references cited therein as an example] while DCM is often necessary to dissolve the chiral catalyst. In most of the cases (entries 1–3 and 6–8, Table 2), 1 mol% of catalyst **4** is enough to promote the reaction in unsatisfactory yields, while 2 mol% of catalyst **4** did not improve the conversion to the desired product **2**. (entry 5, Table 2). It was also demonstrated that higher catalyst loading (entries 4–5, Table 2) required larger volume of dichloromethane with respect to toluene, leading to a decrease of the enantioselectivity of target quaternary amino acid derivative **3**. For these reasons, 1 mol% catalyst was chosen as the optimum loading. Due to the low solubility in toluene, Corey-Lygo catalyst **5** was dissolved in 4:1 mixture of toluene/dichloromethane, to achieve low conversion to compound **2** (entry 4, Table 2). On the other hand, longer residence time did not lead to an improvement of product **2** yield, and this was explained by the possible decomposition of starting imine **1** caused by long contact time with the strong base (entries 2 and 3, Table 2). Best results were obtained by using 3 or 4 CSTR units in line (entries 5–7, Table 2). Higher reaction mixture concentration may trigger precipitation of the catalyst, uneven flow of organic phase through the system and therefore lower conversion to the benzylated compound **2** (entry 8, Table 2).

The ee was determined only on the product obtained with the highest conversion (entry 6, Table 2). Acidic hydrolysis of imine **2** was done under batch conditions using citric acid in THF, to afford stable amino ester **3** after treatment with a base. Under the best reaction conditions, 1 mol% catalyst **4**, 1.4 equiv. BnBr, toluene:DCM 14:1, 3 CSTR units, 80 min residence time (entry 6, Table 2), aminoester **3** was isolated in 20% yield (40% conversion by ^1^H-NMR on product **2** Supplementary Material) and 91% ee, with a slightly better enantioselectivity than in batch (compare entry 6, Table 2 vs. entry 1, Table 1). 

It is noteworthy to underline that the continuous flow system allowed the isolation of the final product **3** without the need of intermediate workup, thus greatly simplifying the overall procedure. 

### 2.3. Solid-Liquid Enantioselective Phase Transfer Benzylation of L-alanine Imine in Flow

Solid-liquid phase transfer conditions imply the use of a solid base to deprotonate the alanine imine. We investigated this methodology using a packed-bed reactor filled with the solid base.

**Figure 3:**
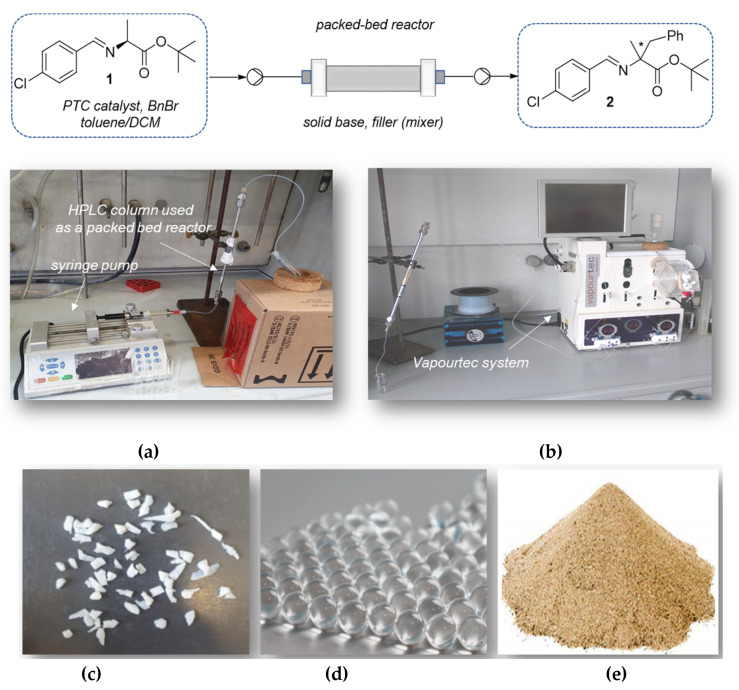
Solid-liquid phase transfer benzylation in packed—bed reactor, (**a**) reaction mixture dosing by syringe pump, (**b**) reaction mixture dosing by Vapourtec pump, (**c**) PTFE boiling stones, (**d**) glass beads or (**e**) sand as inert material.

The setup is illustrated in Figure 3: a standard stainless steel HPLC column (different dimensions depending on the reaction scale), equipped with frit and endcaps from both sides, was filled with the solid base (grounded mixture of KOH and K_2_CO_3_ or CsOH·H_2_O as a solid) and additional inert solids (sand, PTFE boiling stones or glass beads, see Figure 3c–e). The inert material is necessary to enhance the mixing of the two phases inside the reactor, and also to decrease the pressure inside the column. Without the fillers, high pressure is generated inside the reactor due to the very small particles of the solid base, leading to clogging of the reactor. The organic phase (a solution of imine **1**, benzyl bromide and phase transfer catalyst in toluene/DCM) is delivered either by a syringe pump (Figure 3a) or a Vapourtec pump (Figure 3b), into the reactor. 

Selected results of our investigations using syringe pump as a delivery system are reported in Table 3. Also in this case, the ee was determined only on the product obtained with the highest conversion (entry 5, Table 3). A higher loading of phase transfer catalyst **4** did not improve the conversion to product **2**, under the same flow rate (entry 2 vs. entry 3, Table 3). With catalyst **5**, that usually works at higher catalyst loading (see for example [13] and references cited therein) higher volume of dichloromethane was required due to the low solubility of the catalyst in toluene (entries 4–5, Table 3). Comparable conversion to product **2** was observed in 40 min and 66 min residence time. As noticed before in liquid-liquid phase transfer benzylation, these experiments also provide clear evidence about the influence of solvents on the outcome of the reaction. Higher content of toluene compared to dichloromethane can lead to catalyst precipitation inside the reactor, thus leading to irregular reaction mixture flow distribution. Nevertheless, catalyst **5** led to the formation of product **2** in higher conversion although with a lower ee (52% ee of aminoester **3**).

Selected results of our experiments of solid-liquid catalysis in packed-bed reactor using Vapourtec equipment as a delivery system are reported in Table 4. Two HPLC columns of different sizes were used to prepare suitable reactors: one column L 25 cm × OD 6 mm × ID 4.6 mm (entries 1 and 2, Table 4) and one column L 30 cm × OD 7.8 mm (entry 3, Table 4); this last column allowed the possibility of using larger amount of starting material (up to 700 mg of starting imine **1**). Glass beads were used as inert material, enhancing mixing of reagents with solid base inside the reactor. The same flow rate was applied in all the experiments (entries 1–3, Table 4), however the residence time differed depending on reactor dimensions and solid base loading (entry 1–2 vs. entry 3).

1 mol% of catalyst **4** in the presence of strong base CsOH·H_2_O and toluene as the only solvent, was sufficient to promote benzylation of imine **1** in 47% conversion to intermediate **2**, in 10 min residence time only (entry 1, Table 4). It was possible to lower the reaction temperature to 0 °C by placing the packed-bed reactor (the filled HPLC column) vertically into an ice bath: this allowed to improve the enantioselectivity of the reaction in flow up to 93% ee of the final amino ester **3** with respect to 87% ee in batch (entry 4, Table 1). These conditions were the correct compromise to achieve satisfactory conversion with high enantioselectivity. For instance, when using high concentration of the reaction mixture, it was necessary to use DCM only as a solvent to ensure the complete dissolution of catalyst **5**: it was then possible to reach full conversion after 30 min residence time at ambient temperature, at the expense of the ee (56% ee of aminoester **3**, as in batch, entry 3, Table 4). When decreasing the temperature to −15 °C catalyst **5** afforded 60% conversion to product **2** and 72% ee of product **3** after 15 min residence time using toluene:DCM 1.6: 1 (entry 2, Table 4). 

Even in this case, the overall procedure to isolate the final product **3** was largely simplified: the excess base remained in the packed-bed reactor, so at the end of the reaction, the solvent was evaporated to afford product **2** that was then subjected to imine hydrolysis in batch to give product **3** after chromatographic purification (see Supplementary Materials for further details). 

### 2.4. Productivity and Space-Time Yield

In order to fairly compare the batch and the flow phase transfer benzylation of imine **1**, we calculated two metrics that are commonly used to compare different reactors: productivity and space -time yield (STY). Productivity is expressed as mmol of product in the time unit, while STY is commonly used to compare reactors of different size, since it is expressed as mmol of product in the time unit in the volume unit. The calculation is reported in Table 5. The productivity of liquid-liquid continuous flow phase transfer benzylation (Table 2, entry 6) was better than the batch reaction, approximately the double, although the conversion was less than the half. However, there was no improvement in space-time yield compared to batch procedure. 

On the other hand, solid-liquid phase transfer benzylation in packed-bed reactor proved to be strongly superior to batch process, both in terms of productivity and space-time yield. Even with lower conversion, the shorter residence time and the smaller reactor volume guaranteed higher productivity and STY. For example, in the case of the flow reaction of entry 1, Table 4, half of the yield led to a significant ×17 increase in productivity and ×190 in STY with respect to the best result in batch (entry 3, Table 1). In case of entry 2, Table 4, this improvement appears even more marked, with ×25 increase in productivity and ×435 in STY with respect to the best result in batch (entry 3, Table 1). This comparison showed how superior the solid-liquid phase transfer benzylation of imine **1** in packed-bed reactor is with respect to classic phase transfer catalysis in the flask.

## 3. Materials and Methods

### 3.1. Materials and Equipment

Liquid-liquid catalytic phase transfer benzylation of L-alanine imine: all experiments were conducted in continuous stirred tank reactor at room temperature and two phases were separated by membrane separator (Zaiput separation device) at the end of the process. The first two volumes were discarded before reaching the steady state. The suitable membrane to separate two immiscible phases was PTFE hydrophilic membrane (p/n: IL-2000-S10). *f*Reactor (cascaded continuous stirred tank reactor) which may contain 5 CSTR units in line, located onto a metal baseplate and placed on conventional laboratory hotplate-stirrer, was used for our experiments [28]. Standard PTFE tubing (1/8” OD (outer diameter), 1/16 ID (inner diameter)) were used to connect CSTR modules. 

Solid-liquid catalytic phase transfer benzylation was performed in stainless steel HPLC columns (different dimensions depending on the reaction scale) equipped with frit and endcaps from both sides are filled with solid base (grounded mixture of KOH and K_2_CO_3_ or CsOH·H_2_O as a solid) and additional inert solids (sand, PTFE boiling stones or glass beads). Different dimensions HPLC columns were used (L15 cm × ID 4.6 mm; L25 cm × ID 4.6 mm or L30 cm × ID 7.8 mm). The reaction mixture (solution of imine, benzyl bromide and phase transfer catalyst in toluene/dichloromethane) was delivered to packed-bed reactor by Vapourtec E-series continuous flow reactor or Chemyx Fusion 200 modular two-channel syringe pump.

Dry solvents were purchased and stored under nitrogen over molecular sieves (bottles with crown caps). Imine of L-alanine **1** was synthetized according to literature procedure [29].

(11b*S*)-4,4-dibutyl-2,6-bis(3,4,5-trifluorophenyl)-4,5-dihydro-3H-dinaphtho [2,1-c:1′,2′-e] azepinium bromide (Maruoka catalyst **4**) was purchased from Strem Chemicals and was used without further purification.

*O*-allyl-*N*-(9-anthracenylmethyl)cinchonidinium bromide (Corey-Lygo catalyst **5**) was prepared according to literature procedure [30].

### 3.2. Methods

#### 3.2.1. Liquid-Liquid Phase Transfer Benzylation in Flow

*Representative procedure for entry 6 Table 2*: 0.18 mmol (1.0 equiv., 50 mg) of the starting imine **1**, 0.25 mmol (1.4 equiv., 44.5 mg, 0.03 mL) of benzyl bromide and 1.35 mg (1 mol%) of catalyst **4** (entry 6, Table 2) were dissolved in 7.5 mL of toluene/DCM 14:1 and placed in a 10 mL SGA gas tight syringe. 50% aq. sol. of KOH (15 mL) was placed in another SGA gas tight syringe (25 mL). Both syringes are connected to the different syringe pumps. The flow rate of the 50% aq. sol. of KOH was 0.05 mL/min and the flow rate of the organic phase was 0.025 mL/min.

Three CSTR units were used in line. The residence time was calculated according to the reactor volume and flow rate. Calculated residence time is 80 min. Zaiput membrane separator was connected to the last CSTR unit. The organic and water phase were directly separated inside the membrane separator.

The volume of 3 CSTR units is 5.4 mL. The volume of the tube which connects 3 CSTRs is 2 × 0.2 mL (7.5 cm length, od = 1.58 mm, r = 0.58 mm). The volume of the tube at the output is 0.2 mL (the same length like between two CSTR units).

After separation of water/organic phases inside membrane separator, organic phase was collected, solvent was removed under reduced pressure and conversion to the benzylated product **2** was analyzed by ^1^H-NMR in CDCl_3_.

#### 3.2.2. Solid-Liquid Phase Transfer Benzylation in Flow

*Representative procedure for entry 5 Table 3*: In a 25 mL round bottom flask, 0.746 mmol (1.0 equiv., 200 mg) of the starting alanine imine **1**, 1.5 mmol (2.0 equiv., 0.18 mL) of benzyl bromide and 45 mg (10 mol%) of catalyst **5** was dissolved in 6.8 mL of toluene/dichloromethane 1:2.4. The reaction mixture was stirred for 15 min at room temperature, and it was placed in the glass SGA syringe (10 mL). The empty HPLC metal column equipped with endcaps and porous metal frits (L 15 cm × OD 6 mm × ID 4.6 mm) was filled with the mixture of solid bases (420 mg of KOH (10 equiv.), 1 g of K_2_CO_3_ (10 equiv.) and 2 g of sand (~3.4 g of solid inside the column). The reaction mixture inside SGA syringe was delivered by syringe pump into the reactor (HPLC column filled with solid base and sand).

The total volume of the packed-bed reactor was 1.05 mL and the corresponding residence time for the flow rate of 0.015 mL/min was 66 min. The column was positioned vertically, and the solution goes from the bottom up through the column. Each volume of the reaction mixture was collected at the reactor output, solvent was removed under reduced pressure and conversion to the benzylated product **2** was analyzed by ^1^H-NMR in CDCl_3_.

In both cases, after benzylation, relevant collected volumes were dissolved in 2 mL of THF, and 4 mL of 0.5 M aq. sol. of citric acid was added to the solution. The reaction mixture was stirred in a flask, at room temperature for 8 h. After the cleavage completion, the reaction was stopped and THF was removed under reduced pressure. The water phase was washed with cyclohexane (2 × 10 mL). Phases were separated in separatory funnel and water phase was basified using solid K_2_CO_3_ until pH = 10. Basified water layer was extracted with Et_2_O (3 × 15 mL). Organic phases were combined, washed with brine (30 mL), dried over Na_2_SO_4_, filtered, and removed on rotary evaporator. Target amino ester **3** was isolated as a pale-yellow or yellow oil, on which the enantioselectivity was determined.

#### 3.2.3. Analytical Methods

Reactions were monitored by analytical thin-layer chromatography (TLC) using silica gel glass plates (0.25 mm thickness) and visualized using UV light. 1H-NMR spectra were recorded on spectrometers operating at 300 MHz (Bruker Avance 300). Proton chemical shifts are reported in ppm (δ) with the solvent reference relative to tetramethylsilane (TMS) employed as the internal standard (CDCl_3_ δ = 7.26 ppm). ^1^H-NMR spectra were recorded in CDCl_3_, at room temperature. Enantiomeric excess determinations were performed on Agilent 1100 series HPLC, on chiral stationary phase (for the target amino ester **3**, Chiralpak IA, eluent: *n*-hexane/isopropanol 95:5 or Chiralpak AD, eluent: *n*-hexane/isopropanol 95:5, flow rate 1 mL/min).

## 4. Conclusions

In conclusion, the first example of stereoselective catalytic phase transfer synthesis of quaternary amino acid derivatives in continuous flow was reported. The work showed how the use of continuous flow methodologies allowed to achieve satisfactory reactivity of more sterically hindered L-alanine imine to the synthesis of amino acids featuring a quaternary stereocenter. Maruoka catalyst **4**, in combination with toluene as a solvent and low temperature, allowed to isolate the desired aminoester **3** in moderate yield but high ee (up to 93%). Solid-liquid reaction conditions in packed-bed reactor guaranteed much superior performances in terms of productivity and STY with respect to the batch reaction. It was found that the use of a continuous flow system allowed the isolation of the final product **3** without the need of intermediate workup, as requested in batch, thus greatly simplifying the overall procedure. Based on reported findings, we expect that solid-liquid enantioselective phase transfer catalysis in packed-bed reactor will become a popular methodology for the synthesis of enantioenriched amino acids featuring a quaternary stereocenter.

## Figures and Tables

**Table 1:** Preliminary experiments in batch.

Entry	Catalyst	Base	Toluene:DCM Ratio	BnBr (equiv.)	Temperature (°C)	Reaction Time Benzylation (h) ^1^	Yield 3 (%) ^2^	ee 3 (%) ^3^
1	4 (1 mol%)	50% KOH	3:1	1.5	25	28	38	89
2	5 (5 mol%)	KOH/K_2_CO_3_ 5 equiv./5 equiv.	toluene	1.1	0	3	47	52
3	5 (5 mol%)	KOH/K_2_CO_3_ 5 equiv./5 equiv.	1:1	1.1	0	3	62	51
4	4 (1 mol%)	CsOH·H_2_O 5 equiv.	toluene	1.5	0	3	67	87
5	4 (1 mol%)	KOH/K_2_CO_3_ 5 equiv./5 equiv.	toluene	1.5	0	3	59	90

^1^ Refers to phase transfer benzylation (quantitative conversion to compound **2**). ^2^ Isolated yields. ^3^ Determination of ee% was done by HPLC on chiral stationary phase on product **3**.

**Table 2:** Liquid-liquid asymmetric phase transfer benzylation of L-alanine imine **1** in flow in CSTR.

Entry	Catalyst	BnBr (equiv.)	Toluene:DCM Ratio	C op (mol/dm^3^)	Flow Rate-op ^1^ (mL/min)	Flow Rate-wp ^2^ (mL/min)	CSTR Units (number)	Residence Time (min)	^1^H-NMR Conversion to Product 2 (%) ^3^
1	4 (1 mol%)	1.4	14:1	0.024	0.01	0.02	1	60	13
2	4 (1 mol%)	1.5	14:1	0.024	0.0025	0.0025	1	395	15
3	4 (1 mol%)	2.5	14:1	0.024	0.01	0.01	2	220	20
4	5 (10 mol%)	1.4	4:1	0.025	0.1	0.2	3	20	10
5	4 (2 mol%)	1.4	4:1	0.025	0.1	0.2	3	20	37
6	4 (1 mol%)	1.4	14:1	0.025	0.025	0.05	3	80	40, (91% ee) ^4^
7	4 (1 mol%)	1.4	14:1	0.025	0.025	0.05	4	106	38
8	4 (1 mol%)	3	3.6:1	0.2	0.03	0.03	2	64	23

^1^ Flow rate of organic phase (first syringe—solution of imine, benzyl bromide and phase transfer catalyst in toluene/DCM). ^2^ Flow rate of water phase (second syringe—50% aqueous solution of KOH). ^3 1^H-NMR conversion refers to average values of collected volumes (except first two to achieve steady state). ^4^ Determination of ee% was done by HPLC on chiral stationary phase on product **3**.

**Table 3:** Solid-liquid asymmetric phase transfer benzylation of L-alanine imine **1** in packed-bed reactor-reaction mixture delivered by syringe pump (Figure 3a).

Entry ^1^	Catalyst	Toluene:DCM Ratio	Inert Material	Flow Rate-op ^2^ (mL/min)	Residence Time (min)	Solid Base (equiv.)	^1^H-NMR Conversion to the Product 2 (%) ^3^
1	4 (1 mol%)	8.5:1	sand	0.025	40	KOH/K_2_CO_3_ (15/15)	19
2	4 (1 mol%)	8.5:1	PTFE b. s.	0.025	40	KOH/K_2_CO_3_ (10/10)	35
3	4 (5 mol%)	5.2:1	PTFE b. s.	0.025	40	KOH/K_2_CO_3_ (10/10)	28
4	5 (10 mol%)	1:2.4	sand	0.025	40	KOH/K_2_CO_3_ (10/10)	63
5	5 (10 mol%)	1:2.4	sand	0.015	66	KOH/K_2_CO_3_ (10/10)	65, (52% ee) ^4^

^1^ Reactions were carried out with 1 equiv. of imine **1** (0.1 M solution of reaction mixture) and 2 equiv. of benzyl bromide. ^2^ Flow rate of reaction mixture (solution of imine, benzyl bromide and phase transfer catalyst in toluene/DCM). ^3 1^H-NMR conversion refers to average values of collected volumes (firs two volumes discarded to reach the steady state). ^4^ Determination of ee% was done by HPLC on chiral stationary phase on product **3** after cleavage of imine in batch with citric acid and basification with aq. sol. of NaOH, (23% yield, 52% ee).

**Table 4:** Solid-liquid asymmetric phase transfer benzylation of L-alanine imine **1** in flow using packed-bed reactor-reaction mixture delivered by Vapourtec system (Figure 3b).

Entry ^1^	Catalyst	BnBr (equiv.)	Toluene:DCM Ratio	Temperature (°C)	C of op (mol/dm^3^)	Residence Time (min)	Solid Base (equiv.)	^1^H-NMR Conversion to the Product 2 (%) ^2^	ee% of Product 3 ^3^
1	4 (1 mol%)	1.5	toluene	0	0.2	10	CsOH·H_2_O (10)	47	93
2	5 (5 mol%)	1.2	1.6:1	−15	0.3	15	KOH/K_2_CO_3_ (6/6)	60	72
3	5 (10 mol%)	1.2	DCM	25	0.35	30	KOH/K_2_CO_3_ (45/45)	95	56

^1^ The flow rate of reaction mixture—0.1 mL/min. Glass beads were used as an inert material. ^2 1^H-NMR conversion refers to average values of collected volumes (except first two to achieve steady state). ^3^ Determination of ee% was done by chiral HPLC, on a target amino ester **3** after cleavage of imine **2** in batch with citric acid, basification with aq. sol. of NaOH and extraction. Isolated yield of product **3** (entry 1, 15%; entry 2, 20%; entry 3, 30%).

**Table 5:** Comparison of productivity and space-time yield in flow and in batch.

Entry	Method	^1^H-NMR Conversion to the Product 2 (%)	Reactor Volume (mL)	Residence Time (min)	Productivity ^1^ (mmol/h)	Space-Time Yield ^2^ (mmol/mL*h)
Liquid-liquid phase transfer benzylation in CSTR						
Table 1/Entry 1 ^3^	batch	>98	5.43	1680	0.027	0.05
Table 2/Entry 6	flow	40	25	333	0.053	0.002
Solid-liquid phase transfer benzylation in packed-bed reactor						
Table 1/Entry 3 ^3^	batch	>98	4	180	0.248	0.062
Table 3/Entry 5	flow	65	1.05	66	0.436	0.415
Table 4/Entry 1	flow	47	0.35	5	4.2	12
Table 4/Entry 2	flow	60	0.23	4.33	6.23	27
Table 4/Entry 3	flow	95	1	30	1.4	1.4

^1^ Productivity: mmol of product **2** (calculated by ^1^H-NMR conversion, average value) divided by the collection time required to collect 0.75 mmol of benzylated product **2**. ^2^ Space-time yield: productivity divided by reactor volume. ^3^ In batch process to get 0.75 mmol of product **2**.

## Data Availability

Not applicable.

## Supplementary Materials

The following are available online at: https://www.mdpi.com/article/10.3390/molecules28031002/s1. Pictures of equipment; experimental procedures; copies of ^1^H-NMR spectra; copies of HPLC traces [11,14,15,26,28,29,30,31].

## Sample Availability

Samples of the compound **3** are available from the authors.
