# Supplementary material for: Laboratory Scale Continuous Flow Systems for the Enantioselective Phase Transfer Catalytic Synthesis of Quaternary Amino Acids
Source: Molecules. 2023 Jan 19;28(3):1002. doi: 10.3390/molecules28031002 (PMC9920360; doi:10.3390/molecules28031002)
Supplement: Supplementary file 1 [file molecules-28-01002-s001.zip › molecules-2144566-supplementary.pdf]

Supporting Information

Laboratory Scale Continuous Flow Systems for the Enantioselective Phase Transfer Catalytic Synthesis of Quaternary Amino Acids

Milena Krstić[1], Sergio Rossi[1], Miguel Sanz[2] and Alessandra Puglisi[1 *]

[1] Dipartimento di Chimica, Università degli Studi di Milano - Via Golgi 19, 20133 Milano, Italy

[2] Taros Chemicals, GmbH & Co. KG Emil-Figge-Str 76a, 44227 Dortmund Germany

* Correspondence: alessandra.puglisi@unimi.it

Table of Contents

1. General Information 2
2. Description of Equipment 2
3. Experimental Procedures 4
   3.1 In batch phase transfer benzylation of L-alanine imine 4
   3.2 Liquid-liquid phase transfer benzylation of glycine imine in flow 5
   3.3 Liquid-liquid phase transfer benzylation of L-alanine imine in flow 6
   3.4 Solid-liquid phase transfer benzylation of L-alanine imine in flow 7
4. References 9
5. Copies of HPLC traces and ^{1}H NMR 10
   5.1 Copies of ^{1}H NMR spectra – compound 1, 2 and 3 10
   5.2 Copies of ^{1}H NMR spectra – phase transfer benzylation of glycine imine in flow, compound 6 and 7 11
   5.3 Copies of ^{1}H NMR spectra – phase transfer benzylation of alanine imine in flow 12
   5.4 Copies of HPLC traces – experiments in batch, Table 1 14
   5.5 Copies of HPLC traces – experiments in flow, Table 2, 3 and 4 16

1. General Information

Dry solvents were purchased and stored under nitrogen over molecular sieves (bottles with crown caps). Reactions were monitored by analytical thin-layer chromatography (TLC) using silica gel glass plates (0.25 mm thickness) and visualized using UV light. Flash chromatography was carried out on silica gel (230-400 mesh). Proton NMR spectra were recorded on spectrometers operating at 300 MHz (Bruker Avance 300). Proton chemical shifts are reported in ppm (δ) with the solvent reference relative to tetramethylsilane (TMS) employed as the internal standard ($CDCl_3$ δ = 7.26 ppm). ^{1}H NMR spectra were recorded in $CDCl_3$, at room temperature. Enantiomeric excess determinations were performed on Agilent 1100 series HPLC, on chiral stationary phase. Inorganic bases were always freshly prepared prior to use (dissolution of KOH in water to make 50% KOH aq. solution or fine grinding of solid base KOH/K_2CO_3 in mortar with pestle).

(11b*S*) - 4,4-Dibutyl-2,6-bis (3,4,5-trifluorophenyl)-4,5-dihydro-3H-dinaphtho [2,1-c:1',2'-e] azepinium bromide, Maruoka catalyst **4**, was purchased from Strem Chemicals and used as received.

Starting imine of L-alanine **1** was synthetized according to literature procedure [1].

O-Allyl-*N*-(9-anthracenylmethyl)cinchonidinium bromide, Corey-Lygo catalyst **5,** was prepared according to literature procedure [2].

Benzylated imine of L-alanine **2** and target amino ester **3** are known and spectral data are in accordance with the literature [1,3]. Absolute configuration of the amino acid derivative **3** is established [1,4].

2. Description of Equipment

Pumps

The reaction mixture (solution of imine, benzyl bromide and phase transfer catalyst in toluene/dichloromethane) was delivered to packed-bed reactor by Chemyx Fusion 200 modular two-channel syringe pump (1) or Vapourtec E-series continuous flow reactor (2).

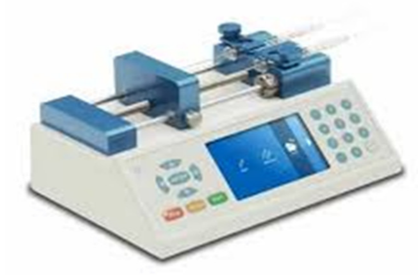

1

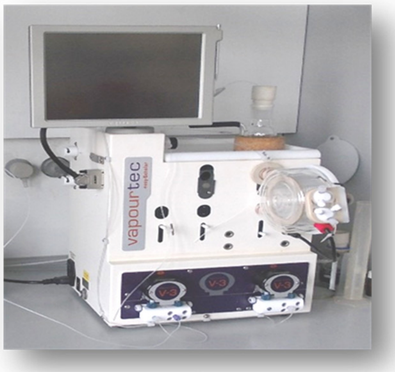

2

Packed-Bed reactor:

Different dimensions stainless steel HPLC columns (L15 cm x ID 4.6 mm; L25 cm x ID 4.6 mm or L30 cm x ID 7.8 mm) filled with solid base and packing material (sand, PTFE boiling stones or glass beads) were used as packed-bed reactor (3).

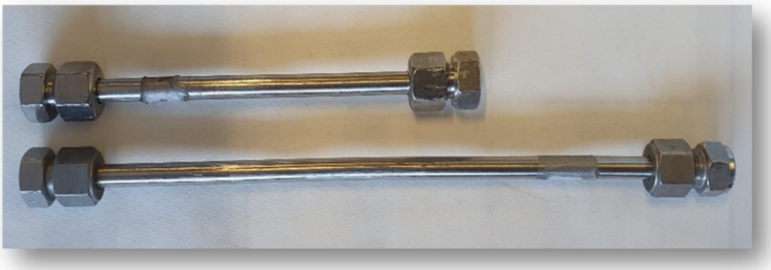

3

Syringes and Zaiput membrane separator:

SGE gas-tight syringes (10 mL and 25 mL) were used to pump reaction mixtures in the reactor (4). In the liquid-liquid phase transfer benzylation Zaiput membrane separator was connected to the last CSTR unit, for the separation of aqueous from organic phase (5).

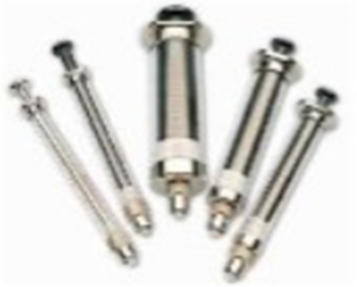

4

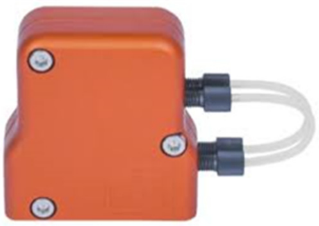

5

Continuous stirred tank reactor-CSTR:

*f*Reactor (cascaded continuous stirred tank reactor) which can contain 5 CSTR units in line, located onto a metal baseplate and placed on conventional laboratory hotplate-stirrer, was used for our experiments [5]. CSTR modules are made of heat and chemical resistant materials, such as high-performance engineering plastic PEEK (polyether ether ketone) and borosilicate glass cover. The volume of one CSTR module is ~1.8 mL. To ensure efficient stirring inside the CSTR units, special PTFE cross stirrer bar was designed, as a part of the whole reactor. Standard PTFE tubing (1/8" OD (outer diameter), 1/16 ID (inner diameter)) are used to connect CSTR modules.

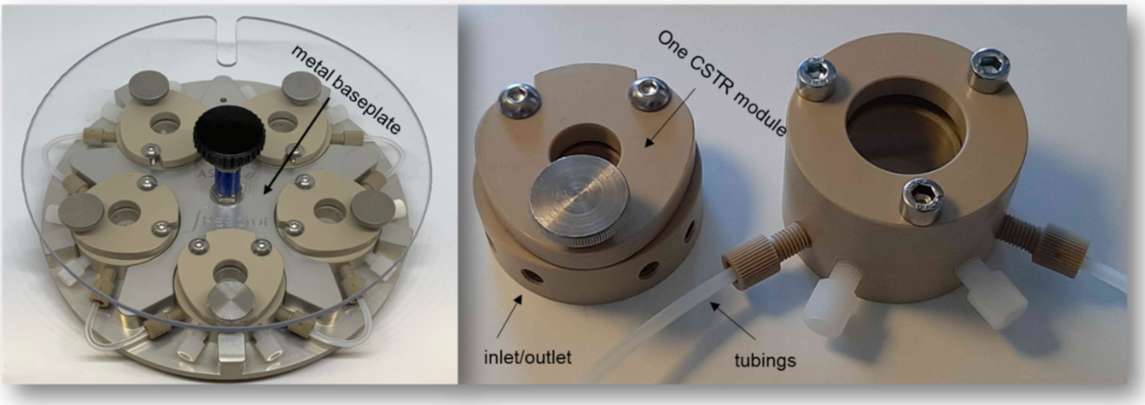

3. Experimental Procedures

General scheme:

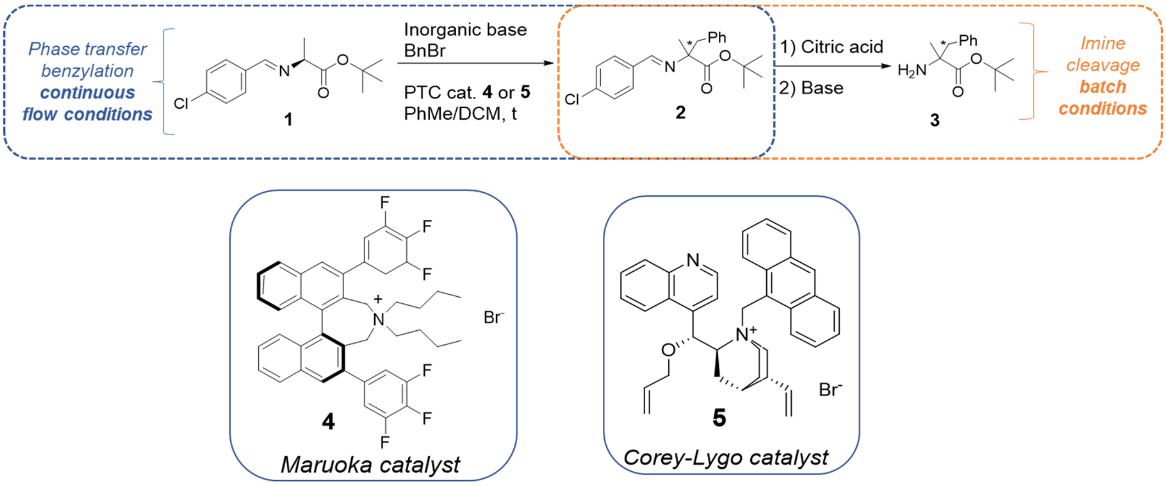

3.1 In batch phase transfer benzylation of L-alanine imine

Experimental procedure for Entry 1, Table 1 (liquid-liquid phase transfer benzylation in batch):

In a two-necked flask with nitrogen inlet, was dissolved 0.3 mmol (80 mg, 1 equiv.) of imine **1** and 0.003 mmol (2 mg, 0.01 equiv.) of Maruoka catalyst **4** in 1.5 mL of toluene (PhMe) and 0.5 mL of dichloromethane (DCM). After dissolution, 0.17 mL (5 equiv.) of 50% aq. KOH was added in the flask.

To a stirred solution (after 5 min) dropwise was added 0.45 mmol (77 mg, 0.05 mL, 1.5 equiv.) of benzyl bromide. Reaction mixture was stirred vigorously (900 rpm) at RT until disappearance of starting imine **1** (monitoring by ^{1}H NMR in $CDCl_3$). After the consumption of starting material (28h), the reaction was stopped, diluted with 15 mL of water and extracted 3 times with 15 mL of PhMe. Organic layers were combined, washed with 30 mL of brine, separated, dried over Na_2SO_4, filtered, and removed under reduced pressure. The residue was dissolved in 1 mL of tetrahydrofuran (THF) and 2 mL of 0.5 M aq. sol. of citric acid. The reaction mixture was stirred on room temperature, until cleavage completion.

The reaction was stopped, THF was removed, and water phase was washed with cyclohexane (2x10 mL). Phases were separated in separatory funnel and water phase was basified by solid K_2CO_3 until pH=10. Basic aqueous layer was extracted with Et_2O (3x15 mL). Organic phases were combined, washed with brine (30 mL), dried over Na_2SO_4, filtered and removed on rotary evaporator.

27 mg of product **3** was isolated as a colorless oil, 38% yield. The enantioselectivity of the compound **3** was determined by chiral HPLC (Chiralpak AD column, eluent: *n*-hexane/isopropanol 95:5, flow rate 1 mL/min (t(major)=6.104 min, t(minor)= 8.827 min, 89% ee).

^{1}H NMR of aminoester **3** (300 MHz, $CDCl_3$) δ ppm: 1.39 (s, 3H), 1.48 (s, 9H), 2.80 (d, 1H), 3.14 (d, 1H), 7.23-7.30 (m, 5H).

Experimental procedure for Entry 5, Table 1 (solid-liquid phase transfer benzylation in batch):

A two-necked flask connected to nitrogen inlet, was charged with 0.37 mmol (100 mg, 1 equiv.) of imine **1**, 0.004 mmol (3 mg, 0.01 equiv.) of Maruoka catalyst **4**, 0.56 mmol (96 mg, 0.07 mL, 1.5 equiv.) of benzyl bromide, 1.86 mmol (105 mg, 5 equiv.) of kalium hydroxide, 1.86 mmol (258 mg, 5 equiv.) of potassium hydroxide, and 1.8 mL of PhMe dried over molecular sieves. The reaction mixture was stirred vigorously at 0°C. After the complete conversion of starting imine **1** to benzylated imine **2** (3h), the reaction was stopped, diluted with 15 mL of water and extracted 3 times with 15 mL of DCM. Organic layers were combined, washed with 30 mL of brine, separated, dried over MgSO4, filtered and removed under reduced pressure. The residue was dissolved in 4 mL of THF and 5 mL of 15% sol. of citric acid monohydrate (pH=2-3). Reaction mixture was stirred at room temperature for 5h, THF was removed, and residue was washed with cyclohexane (15 mL). Phases were separated in separatory funnel; aqueous phase was basified by solid Na_2CO_3 (pH=9) and extracted with DCM (3x15 mL). Organic phases were combined, washed with brine (30 mL), dried over MgSO4, filtered, and removed on rotary evaporator. 52 mg of product **3** was isolated as light-yellow oil, 59% yield. The enantioselectivity of the compound **3** was determined by chiral HPLC (Chiralpak AD column, eluent: *n*-hexane/isopropanol 95:5, flow rate 1 mL/min (t(major)=6.007 min, t(minor)= 8.660 min, 90% ee).

Liquid-liquid or solid-liquid phase transfer benzylation in batch was performed according to the well-known synthetic procedures [4,6,7].

3.2 Liquid-liquid phase transfer benzylation of glycine imine in flow

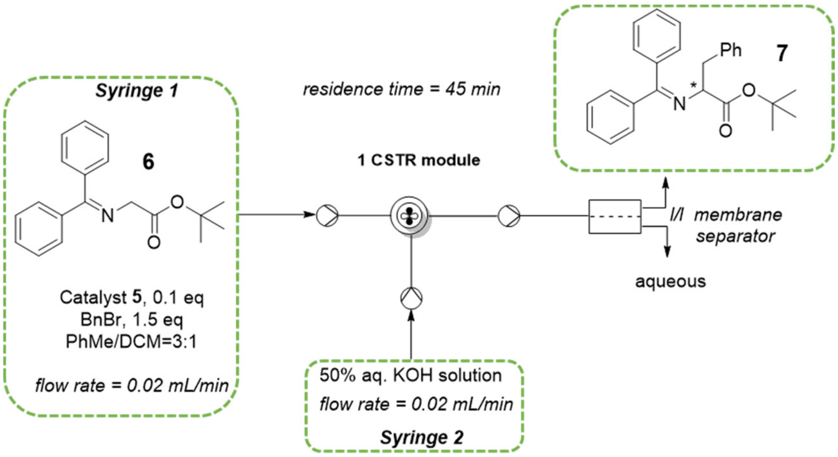

0.203 mmol (60 mg, 1 equiv.) of glycine imine **6**, 0.305 mmol (52 mg, 0.036 mL, 1.5 equiv.) of BnBr and 0.02 mmol (12.2 mg, 0.1 equiv.) of cinchona catalyst **5** was dissolved in 8 mL of PhMe:DCM=3:1, stirred for 5 min in the flask and then placed in a in a HSW plastic syringe (10 mL).

4 g of KOH was dissolved in 8 mL of water (50% aq. sol. of KOH=1:1) and was placed in another HSW plastic syringe (10 mL). Both syringes were connected to the same syringe pump and delivered the flow of 0.02 mL/min (overall 0.04 mL/min). Only one CSTR unit was used.

The residence time calculated for 1 CSTR unit is 45 min. Zaiput membrane separator was connected to the CSTR output. The first 3 volumes were collected, toluene was removed, and the conversion to the product **7** was monitored by ^{1}H-NMR in $CDCl_3$ (~60% conversion to the benzylated imine of glycine).

4th-7th volumes were collected afterwards, solvent was evaporated, and the reaction mixture was checked by ^{1}H-NMR (~80% of conversion to the product **7**). Overall, the representative volumes showed around 80% conversion of starting glycine imine to the product **7**, with the residence time of 45 min (one CSTR unit used).

This experiment serves as a proof of different reactivity between imine of glycine and imine of alanine. Asymmetric PT benzylation done with imine of glycine (more reactive imine) has been reproduced successfully based on the published article [8].

Further cleavage of product **7** was not performed, due to the higher stability of glycine imine **6** compared to alanine imine **1**.

3.3 Liquid-liquid phase transfer benzylation of L-alanine imine in flow

Experimental procedure for Entry 6, Table 2:

0.18 mmol (1.0 equiv., 50 mg) of the starting imine **1**, 0.25 mmol (1.4 equiv., 44.5 mg, 0.03 mL) of benzyl bromide and 1.35 mg (1 mol%) of the Maruoka catalyst **4** were dissolved in 7.5 mL of PhMe/DCM 14:1 and placed in a 10 mL SGA gas tight syringe. 50% aq. sol. of KOH (15 mL) was prepared and transferred in another SGA gas tight syringe (25 mL). Both syringes are connected to the different syringe pumps. The flow rate of the 50% aq. sol. of KOH was 0.05 mL/min and the flow rate of the organic phase was 0.025 mL/min. Three CSTR modules were used in line. The residence time was calculated for 3 CSTR units + tubing between them and between the third CSTR and membrane separator. Calculated residence time is 80 min. Zaiput membrane separator was connected to the last CSTR unit.

The volume of 3 CSTR units is 5.4 mL. The volume of the tube which connects 3 CSTRs is 2x0.2 mL (7.5 cm length, od=1.58 mm, r=0.58 mm). The volume of the tube at the output is 0.2 mL (the same length like between two CSTR units).

After separation of water/organic phases inside membrane separator, organic phase was collected, solvent was removed under reduced pressure and conversion to the benzylated product **2** was analyzed by ^{1}H NMR in $CDCl_3$. Collected volumes (average value showing ~40% conversion to the product **2**) were dissolved in 2 mL of THF and 4 mL of 0.5 M aq. sol. of citric acid was added to the solution. The reaction mixture was stirred at room temperature 8h. After 8h, the reaction was stopped and THF was removed under reduced pressure. The water phase was washed with cyclohexane (2x10 mL). Phases were separated in separatory funnel and water phase was basified using solid K_2CO_3 until pH=10. Basified water layer was extracted with Et_2O (3x15 mL). Organic phases were combined,

washed with brine (30 mL), dried over Na_2SO_4, filtered, and removed on rotary evaporator. Finally, amino ester **3** was isolated in 20% yield, as a pale-yellow oil.

The enantioselectivity of the compound **3** was determined by chiral HPLC (Chiralpak AD column, eluent: *n*-hexane/isopropanol 95:5, flow rate 1 mL/min, t(major)=6.06 min, t(minor)= 8.7 min, 91% ee).

3.4 Solid-liquid phase transfer benzylation of L-alanine imine in flow

Experimental procedure for Entry 5, Table 3:

In a 25 mL round bottom flask, 0.746 mmol (1.0 equiv., 200 mg) of the starting alanine imine **1**, 1.5 mmol (2.0 equiv., 0.18 mL) of benzyl bromide and 45 mg (10 mol%) of the cinchona catalyst **5** was dissolved in 6.8 mL of PhMe/DCM 1:2.4. The reaction mixture was stirred for 15 min at room temperature, and it was placed in the glass SGA syringe (10 mL). Empty HPLC metal column equipped with endcaps and porous metal frits (L 15 cm x OD 6 mm x ID 4.6 mm) was filled with the mixture of solid bases (420 mg of KOH (10 equiv.), 1g of K_2CO_3 (10 equiv.) and 2 g of sand (~3.4 g of solid inside the column). The reaction mixture inside SGA syringe was delivered by syringe pump into the reactor (HPLC column filled with solid base and sand). The total volume of the packed-bed reactor was 1.05 mL and the corresponding residence time for the flow rate of 0.015 mL/min was 66 min. The column was positioned vertically, and the solution goes from the bottom up through the column. Each volume of the reaction mixture was collected at the reactor output, solvent was removed under reduced pressure and conversion to the benzylated product **2** was analyzed by ^1H NMR in $CDCl_3$, showing ~65% conversion to the benzylated product **2**. The cleavage of benzylated imine **2** was performed in batch, as previously described. Subsequently, additional purification on silica gel column chromatography (eluent *n*-hexane/ethyl acetate 1:1) was applied to afford aminoester **3** in 23% yield. The enantioselectivity of the compound **3** was determined by chiral HPLC (Chiralpak AD column, eluent: *n*-hexane/isopropanol 95:5, flow rate 1 mL/min, t(major)=8.15 min, t(minor)= 5.84 min, 52% ee).

Experimental procedure for Entry 1, Table 4:

25 mL flask with a nitrogen inlet, was charged with 400 mg (1.5 mmol, 1.0 equiv.) of imine **1**, 10 mg (0.015 mmol, 0.01 equiv.) of Maruoka catalyst **4** and 383 mg (2.2 mmol, 1.5 equiv.) of benzyl bromide dissolved in 7.5 mL of toluene dried over molecular sieves (C=0.2 M). Stainless steel HPLC column (packed-bed reactor) with endcaps and frits (L 25 cm x OD 6 mm x ID 4.6 mm) was filled with the solid base $CsOH{\cdot}H_2O$ (2.5-2.6 g, ~0.015 mol, ~10 equiv.) and glass (soda-lime) beads (4.5 g). Glass beads were used to reduce high pressure in the system and the packing of $CsOH{\cdot}H_2O$ was done very fast, due to high hygroscopicity. HPLC metal column was placed vertically in plastic graduated cylinder and cooled down to 0 °C in ice/water cooling bath. The reaction mixture from the flask was delivered into packed-bed reactor by pump A of Vapourtec flow system through PTFE tubing. The volume of the PTFE tubes was not considered because the reaction occurs inside the packed-bed reactor.

All fractions were collected after every 10 min (one volume 0.7 mL) with the flow rate of reaction mixture 0.1 mL/min. The reaction was monitored by ^1H NMR in $CDCl_3$ (average conversion ~47%).

After the completion of the flow process, the relevant fractions were used for the acidic cleavage of imine **2** in batch conditions to afford amino ester **3** as a light-yellow oil, ~15% yield. The cleavage of benzylated imine **2** was performed in batch, as previously described.

The enantioselectivity of the compound **3** was determined by chiral HPLC (Chiralpak AD column, eluent: *n*-hexane/isopropanol 95:5, flow rate 1 mL/min, t(major)=6.03 min, t(minor)= 8.7 min, 93% ee.

Experimental procedure for Entry 2, Table 4:

25 mL round bottom flask with a nitrogen inlet, was charged with 700 mg (2.6 mmol, 1.0 equiv.) of alanine imine **1**, 79 mg (0.13 mmol, 0.05 equiv.) of cinchona catalyst **5** and 537 mg (0.89 mmol, 0.37 mL, 1.2 equiv.) of benzyl bromide dissolved in 5.2 mL of toluene and 3.3 mL of dichloromethane (PhMe/DCM 1.6:1). The flask with reaction mixture was cooled down in the cooling bath ice/NaCl (-15 °C) and connected to the Vapourtec continuous flow system.

HPLC metal column (250 mm x 4.6 mm) was filled with the mixture of base KOH/K_2CO_3 1:1 (2.9 g in total (2.06 g of K_2CO_3 and 0.84 g of KOH), ~6 equiv. each) and glass (soda-lime) beads (3.77 g). HPLC metal column was cooled down in the cooling bath ice/NaCl (-15 °C) and attached through PTFE tube (0.8mm x 1.6 mm) and corresponding fittings with the pump A of Vapourtec machine. Fractions were collected after each 15 min (residence time for flow rate 0.1 mL/min) and the total volume of the reactor is 0.8 mL. The reaction was monitored by ^1H NMR in $CDCl_3$, showing ~60% conversion to the product **2**. Toluene was removed from each fraction; residue was dissolved in $CDCl_3$ and analyzed by ^1H NMR. After the completion of the flow process, the relevant fractions were used for the acidic cleavage of imine **2** in batch conditions to afford amino ester **3** in ~20% yield. The cleavage of benzylated imine **2** was performed in batch, as previously described.

The enantioselectivity of the compound **3** was determined by chiral HPLC (Chiralpack IA column, eluent: *n*-hexane/isopropanol 9:1, flow rate 1 mL/min, t(major)=8.33 min, t(minor)= 5.63 min, 72% ee.

Experimental procedure for Entry 3, Table 4:

50 mL round bottom flask with a nitrogen inlet, was charged with 200 mg (0.7 mmol, 1.0 equiv.) of alanine imine **1**, 45 mg (0.07 mmol, 0.1 equiv.) of Corey-Lygo catalyst **5**, 153 mg (0.89 mmol, 0.1 mL, 1.2 equiv.) of benzyl bromide and 2 mL of DCM. The flask with reaction mixture was connected to the continuous flow Vapourtec system. On the other side, HPLC metal column (L 30 cm x 7.8 mm) was filled with the mixture of base KOH/K_2CO_3 1:1 (6.55 g in total, 45 equiv. each) and glass (soda-lime) beads (13 g). HPLC metal column was attached through PTFE tube (0.8mm x 1.6 mm), and corresponding fittings with the pump A of Vapourtec machine. Fractions were collected after each 30 min (residence time for flow rate 0.1 mL/min, reactor volume 1mL). The reaction was monitored by benchtop ^1H NMR (Spinsolve 60 ultra-carbon NMR spectrometer) in reaction solvent, dichloromethane. Four volumes were collected and all of them showed complete conversion of alanine imine **1** to the product **2**. All four fractions were collected, solvent was evaporated, and 94 mg of yellow oil was isolated (product **2**). 94 mg was dissolved in 1 mL of THF and 1 mL of 5% citric acid was added. Reaction mixture was stirred at room temperature for 4h. Solution was diluted with diethyl ether (10 mL) and extracted with water (10 mL). The acid extract (pH=4) was than basified (sat. aq. sol. K_2CO_3) and extracted with ethyl acetate (3x20 mL). The ethyl acetate extracts were then washed with brine, dried over $MgSO_4$, and concentrated under reduced pressure. 34 mg of aminoester **3** was isolated as a pink

oil (~30% yield). The enantioselectivity of the compound **3** was determined by chiral HPLC (RegisPack 25 cm X 4.6 mm 5 Micron column, eluent: *n*-hexane/isopropanol 9:1, flow rate 1 mL/min, t(major)=4.7 min, t(minor)= 4.09 min, 56% ee.

4. References

1. Shirakawa, S.; Yamamoto, K.; Liu, K.; Maruoka, K. Enantioselective Alkylation of 2-[(4-Chlorobenzyliden)Amino]Propanoic Acid *tert*-Butyl Ester: Synthesis of (*R*)-2-Amino-2-Methyl-3-Phenylpropanoic Acid tert-Butyl Ester. *Org. Synth.* **2013**, *90*, 121-129.
2. Corey, E. J.; Noe, M. C. Preparation of *O*-allyl-*N*-(9-anthracenylmethyl)cinchonidinium bromide as a phase transfer catalyst for the enantioselective alkylation of glycine benzophenone imine *tert*-butyl ester: (4*S*)-2-(benzhydrylidenamino)pentanedioic, 1-*tert*-butyl ester-5-methyl ester. *Org. Synth.* **2003**, *80*, 38-45.
3. Jew, S.-S.; Jeong, B.-S.; Lee, J.-H.; Yoo, M.-S.; Lee, Y.-J.; Park, B.-S.; Kim, M. G.; Park, H.-G. Highly Enantioselective Synthesis of α-alkyl-alanines via the Catalytic Phase-Transfer Alkylation of 2-Naphthyl Aldimine *tert*-Butyl Ester by Using *O*(9)-Allyl-*N*(1)-2',3',4'-trifluorobenzylhydrocinchonidinium Bromide. *J. Org. Chem*. **2003**, *68*, 4514-4516.
4. Lygo, B.; Crosby, J.; Peterson, J. A. Enantioselective Alkylation of Alanine-Derived Imines Using Quaternary Ammonium Catalysts. *Tetrahedron Lett.* **1999**, *40*, 8671-8674.
5. https://www.freactor.com/product_freactorCSTR.html
6. O'Donnell, M. J.; Wu, S. A Catalytic Enantioselective Synthesis of α-Methyl Amino Acid Derivatives by Phase-Transfer Catalysis. *Tetrahedron Asymmetry* **1992**, *3*, 591-594.
7. Kitamura, M.; Shirakawa, S.; Maruoka, K. Powerful chiral phase-transfer catalysts for the asymmetric synthesis of α-alkyl- and α,α-dialkyl-α-amino acids. *Angew. Chem. Int. Ed*. **2005**, *44*, 1549 –1551.
8. Mo, Y.; Lin, H.; Jensen, K. F. High-performance miniature CSTR for biphasic C–C bond-forming reactions. *Chem. Eng. J.* **2018**, *335*, 936–944.

5. Copies of HPLC traces and ^{1}H NMR

5.1 Copies of ^{1}H NMR spectra – compound 1, 2 and 3

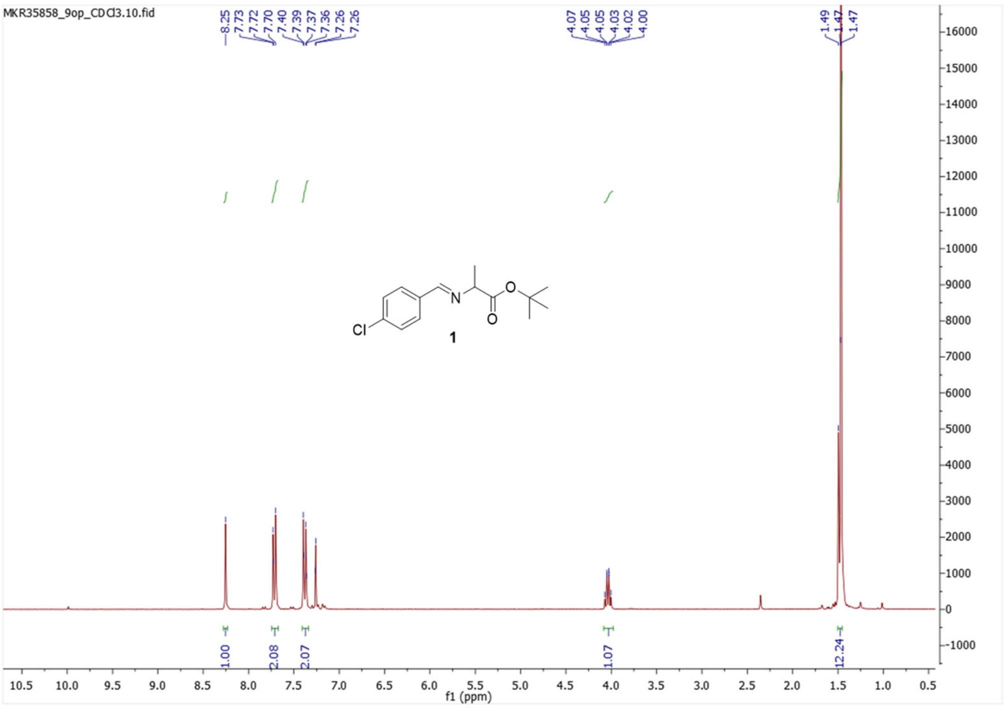

^{1}H NMR of alanine imine **1** in $CDCl_3$

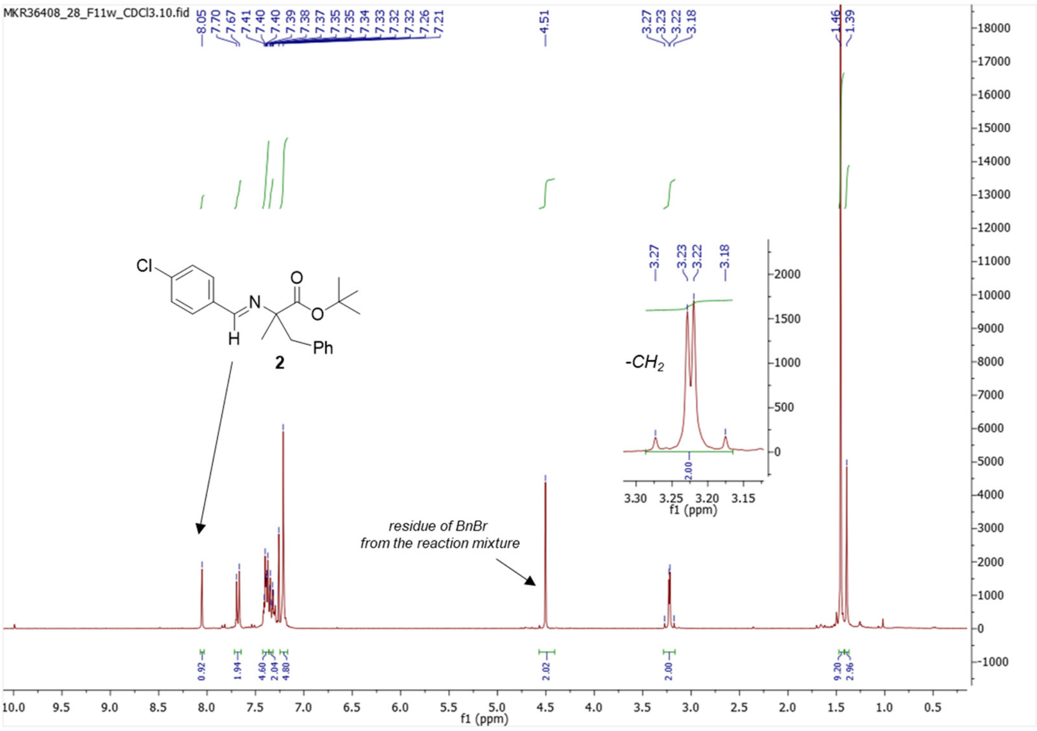

^{1}H NMR of benzylated imine **2** in $CDCl_3$

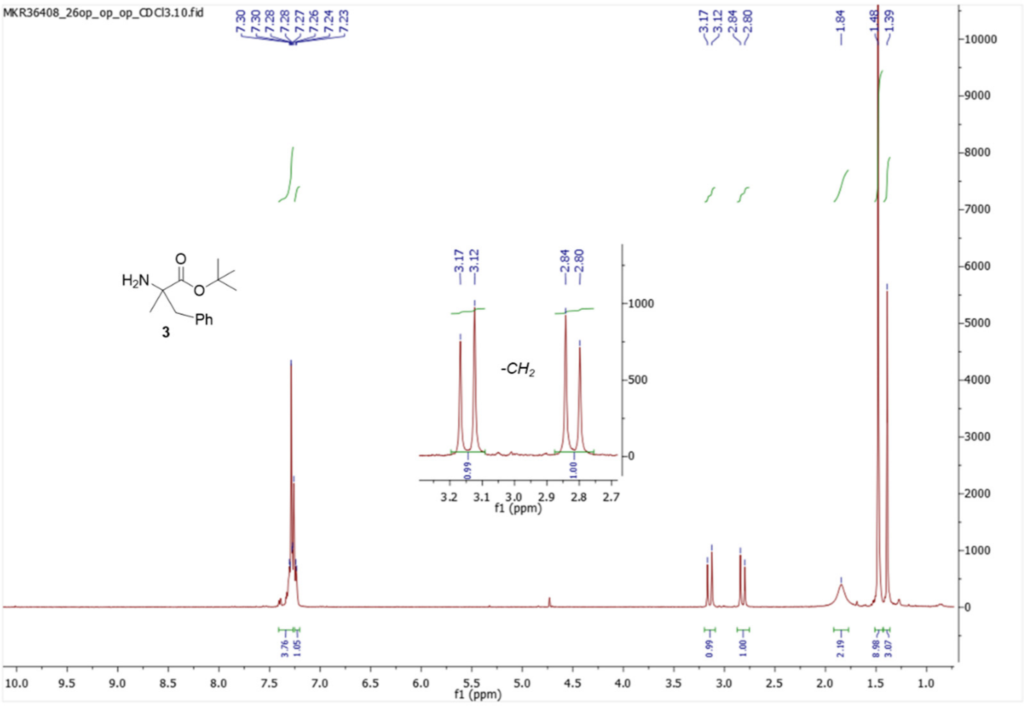

^{1}H NMR of aminoester **3** in $CDCl_3$

5.2 Copies of ^{1}H NMR spectra – phase transfer benzylation of glycine imine in flow, compound 6 and 7

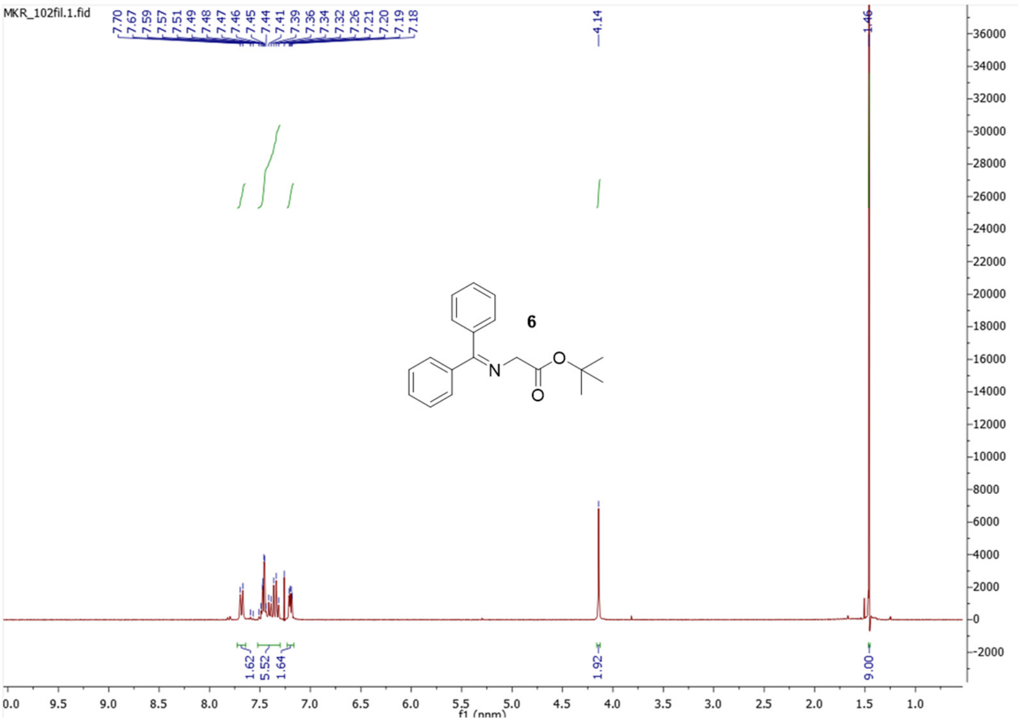

^{1}H NMR of starting glycine imine **6** in $CDCl_3$

Liquid-liquid phase transfer benzylation of glycine imine 6 in continuous stirred-tank reactor - proof of reproducibility:

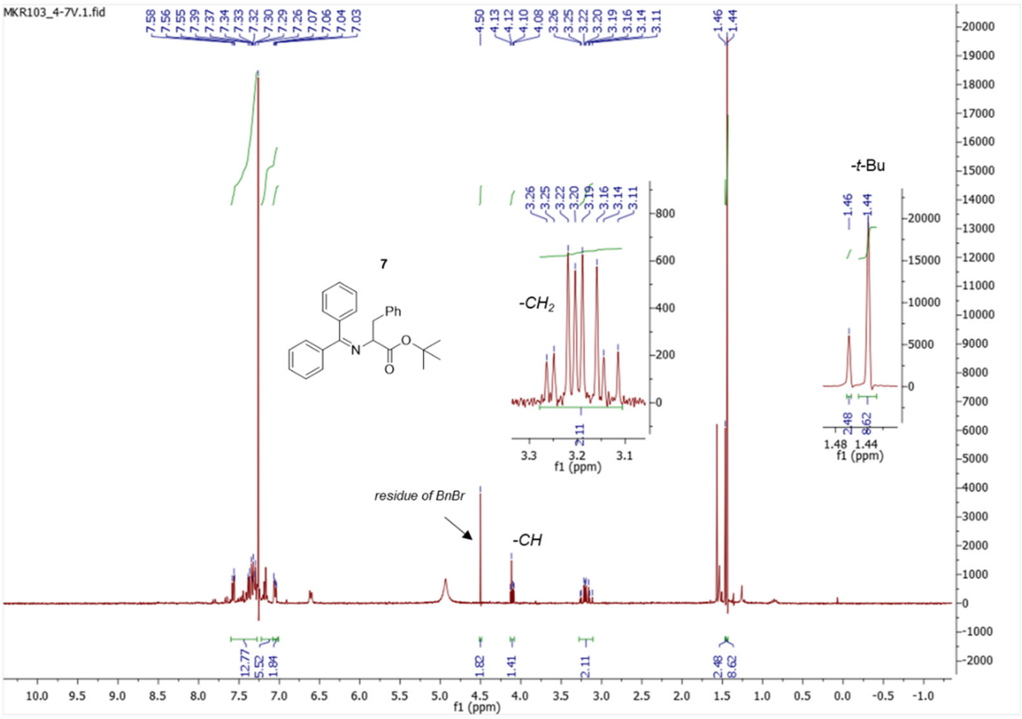

^{1}H NMR conversion of imine **6** to compound **7** (steady state achieved after the 3rd volume)

5.3 Copies of ^{1}H NMR spectra – phase transfer benzylation of alanine imine in flow

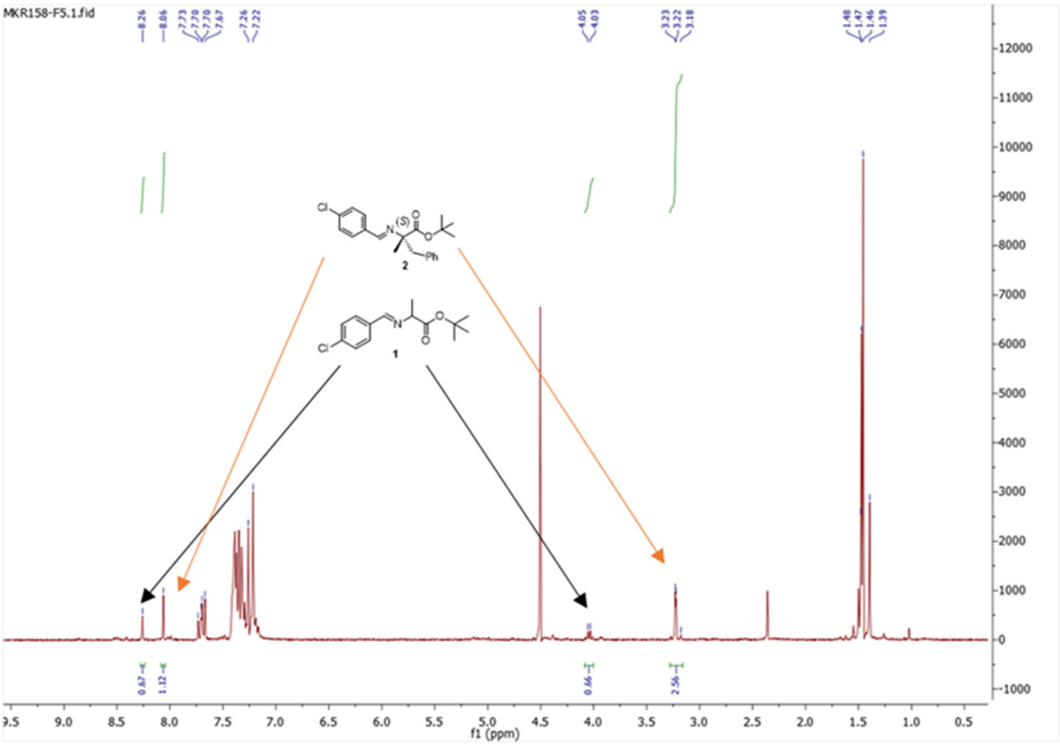

^{1}H NMR conversion of imine **1** to compound **2**/solid-liquid phase transfer benzylation *in continuo* (entry 5, Table 3)

Solid-liquid asymmetric phase transfer benzylation of L-alanine imine 1 in flow using packed-bed reactor (Entry 3, Table 4) – complete conversion to product 2 (b) after deprotonation with solid base KOH/K_2CO_3

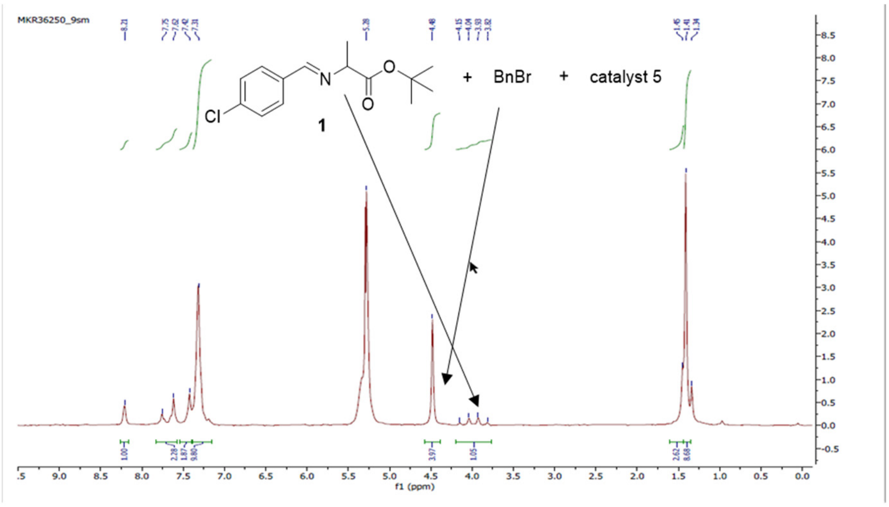

a) ^{1}H-NMR in DCM (60 MHz): Mixture of benzyl bromide, starting imine and the catalyst (before starting the reaction)

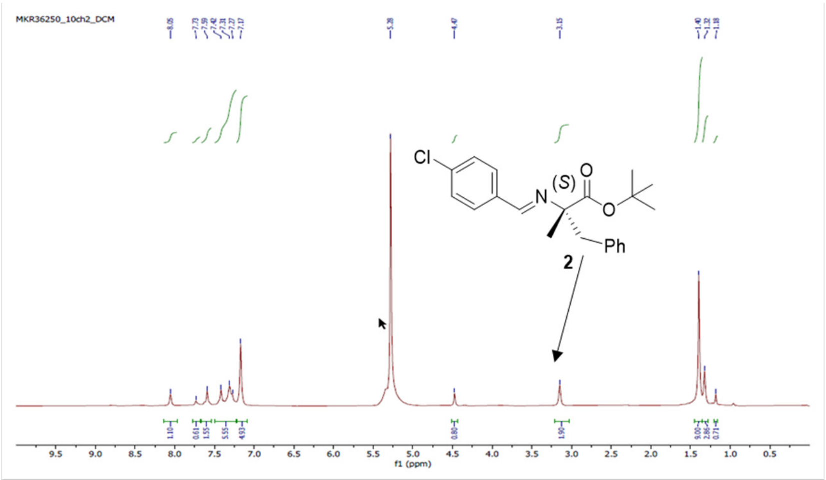

b) ^{1}H-NMR in DCM (60 MHz): PT benzylation in packed-bed reactor - second reaction volume (flow rate 0.1 mL/min)

Reaction mixture monitoring by benchtop ^{1}H NMR (Spinsolve 60 ultra-carbon NMR spectrometer) in DCM, ***before*** (a) and ***after*** (b) PT benzylation in packed-bed reactor

5.4 Copies of HPLC traces – experiments in batch, Table 1

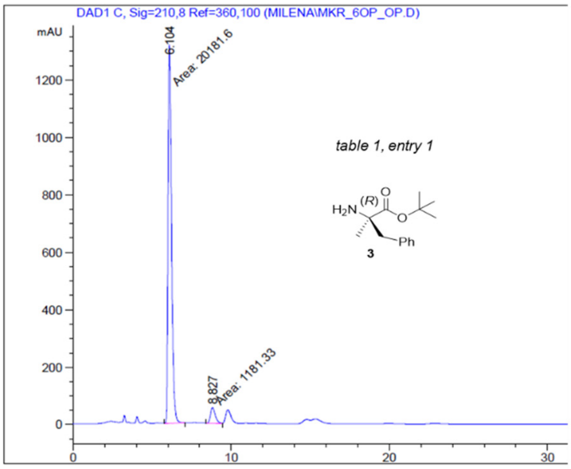

Peak #	RetTime [min]	Type	Width [min]	Area [mAU*s]	Height [mAU]	Area %
1	6.104	MM	0.2550	2.01816e4	1319.27478	94.4702
2	8.827	MM	0.3572	1181.32764	55.11677	5.5298
Totals :				2.13629e4	1374.39155	

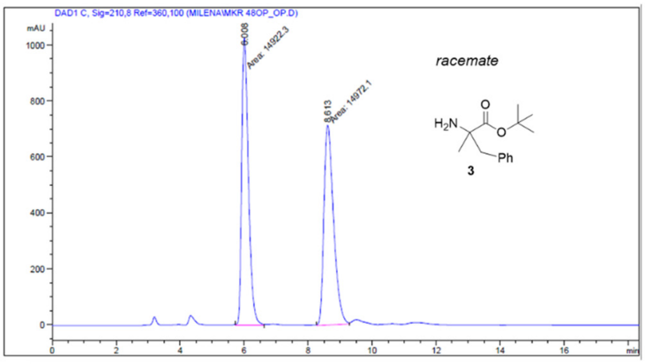

Peak #	RetTime [min]	Type	Width [min]	Area [mAU*s]	Height [mAU]	Area %
1	6.008	MM	0.2420	1.49223e4	1027.78503	49.9168
2	8.613	MM	0.3492	1.49721e4	714.66260	50.0832
Totals :				2.98944e4	1742.44763	

Chiralpak AD column, eluent: *n*-hexane/isopropanol 95:5, flow rate 1 mL/min

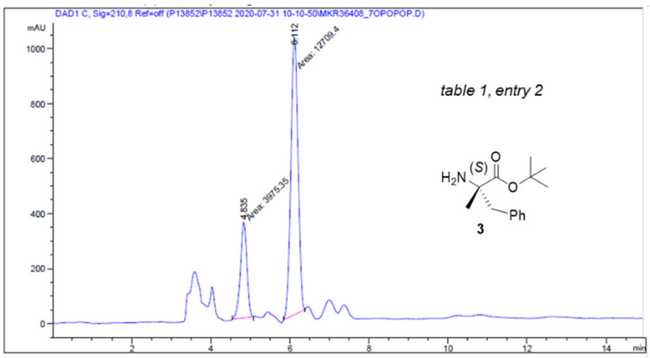

Signal 1: DAD1 C, Sig=210,8 Ref=off

Peak #	RetTime [min]	Type	Width [min]	Area [mAU*s]	Height [mAU]	Area %
1	4.835	MM	0.1905	3975.35352	347.84772	23.8263
2	6.112	MM	0.2101	1.27094e4	1008.31061	76.1737
Totals :				1.66847e4	1356.15833	

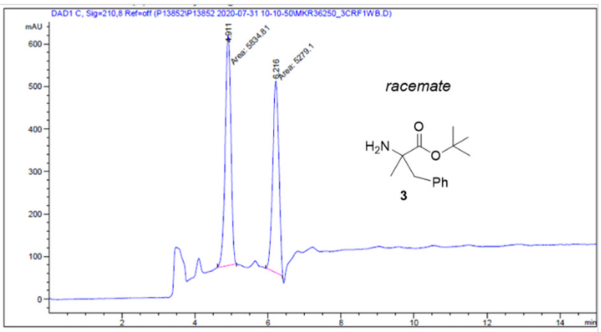

Signal 1: DAD1 C, Sig=210,8 Ref=off

Peak #	RetTime [min]	Type	Width [min]	Area [mAU*s]	Height [mAU]	Area %
1	4.911	MM	0.1797	5834.80811	541.28607	52.5000
2	6.216	MM	0.1954	5279.10498	450.28055	47.5000
Totals :				1.11139e4	991.56662	

RegisPack 25 cm X 4.6 mm 5 Micron column, eluent: *n*-hexane/isopropanol 95:5, flow rate 1 mL/min

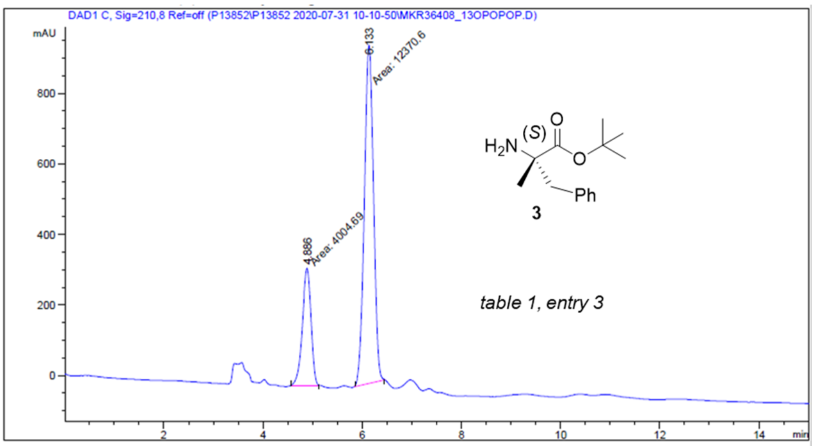

Signal 1: DAD1 C, Sig=210,8 Ref=off

Peak #	RetTime [min]	Type	Width [min]	Area [mAU*s]	Height [mAU]	Area %
1	4.886	MM	0.2007	4004.68604	332.48300	24.4556
2	6.133	MM	0.2147	1.23706e4	960.32092	75.5444
Totals :				1.63753e4	1292.80392	

RegisPack 25 cm X 4.6 mm 5 Micron column, eluent: *n*-hexane/isopropanol 95:5, flow rate 1 mL/min

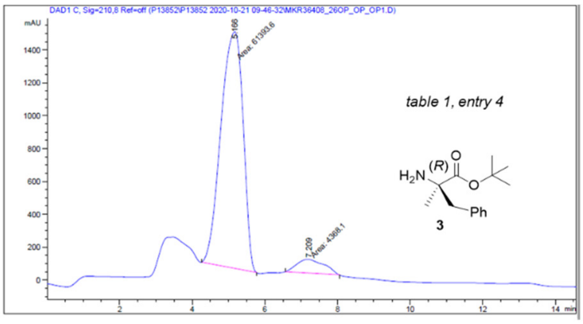

Signal 1: DAD1 C, Sig=210,8 Ref=off

Peak #	RetTime [min]	Type	Width [min]	Area [mAU*s]	Height [mAU]	Area %
1	5.166	MM	0.7092	6.13936e4	1442.79089	93.3577
2	7.209	MM	0.8736	4368.09521	83.33288	6.6423
Totals :				6.57617e4	1526.12377	

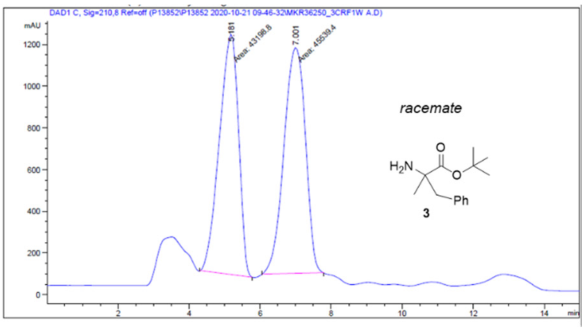

Signal 1: DAD1 C, Sig=210,8 Ref=off

Peak #	RetTime [min]	Type	Width [min]	Area [mAU*s]	Height [mAU]	Area %
1	5.181	MM	0.6243	4.31988e4	1153.23218	48.6812
2	7.001	MM	0.7001	4.55394e4	1084.13599	51.3188
Totals :				8.87382e4	2237.36816	

Chiralpack IA, eluent: *n*-hexane/isopropanol 95:5, flow rate 1 mL/min

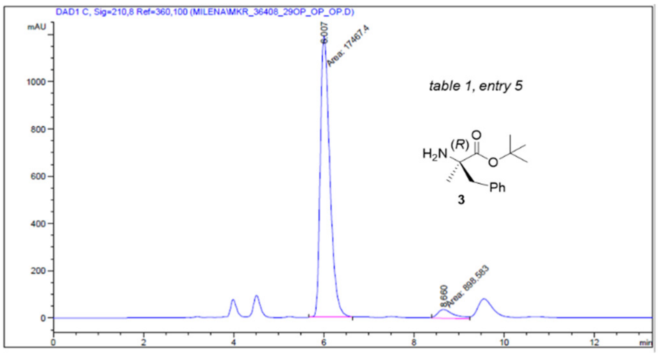

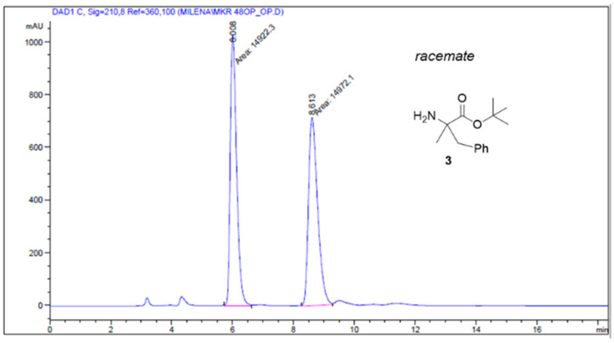

Peak #	RetTime [min]	Type	Width [min]	Area [mAU*s]	Height [mAU]	Area %
1	6.007	MM	0.2446	1.74674e4	1190.14294	95.1074
2	8.660	MM	0.4032	898.58282	37.14561	4.8926
Totals :				1.83660e4	1227.28856	

Peak #	RetTime [min]	Type	Width [min]	Area [mAU*s]	Height [mAU]	Area %
1	6.008	MM	0.2420	1.49223e4	1027.78503	49.9168
2	8.613	MM	0.3492	1.49721e4	714.66260	50.0832
Totals :				2.98944e4	1742.44763	

Chiralpak AD column, eluent: *n*-hexane/isopropanol 95:5, flow rate 1 mL/min

5.5 Copies of HPLC traces – experiments in flow, Table 2, 3 and 4

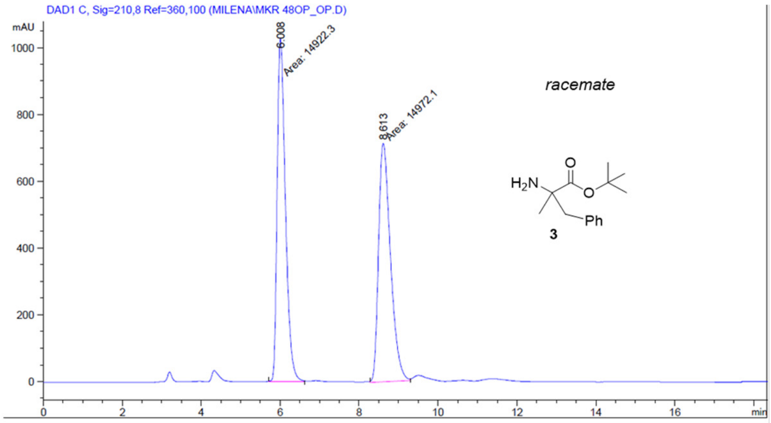

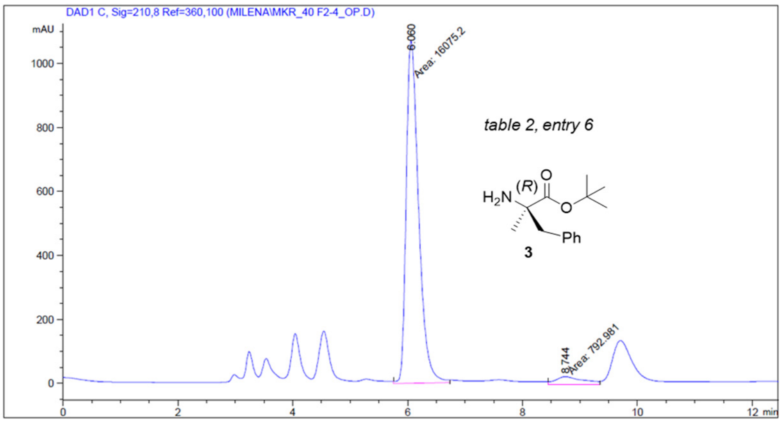

Peak #	RetTime [min]	Type	Width [min]	Area [mAU*s]	Height [mAU]	Area %
1	6.060	MM	0.2503	1.60752e4	1070.56421	95.2990
2	8.744	MM	0.5559	792.98090	23.77531	4.7010

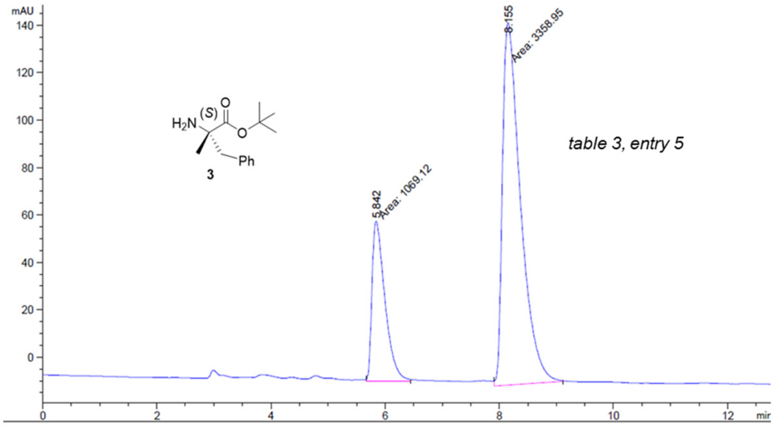

Peak #	RetTime [min]	Type	Width [min]	Area [mAU*s]	Height [mAU]	Area %
1	5.842	MM	0.2638	1069.11768	67.55331	24.1441
2	8.155	MM	0.3662	3358.95386	152.87383	75.8559

Totals : 4428.07153 220.42713

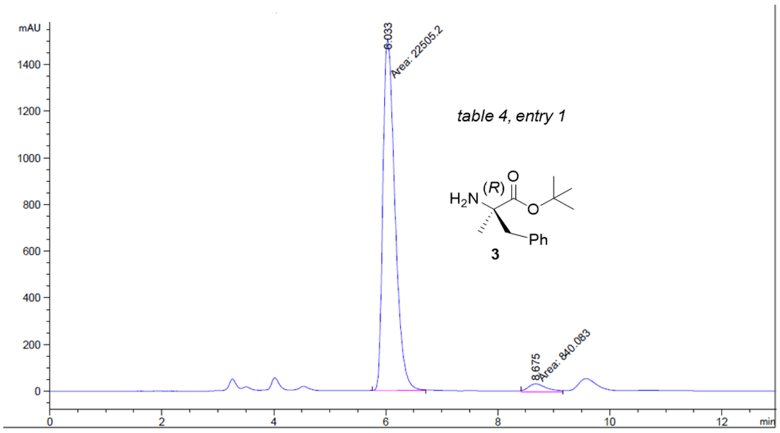

Peak #	RetTime [min]	Type	Width [min]	Area [mAU*s]	Height [mAU]	Area %
1	6.033	MM	0.2494	2.25052e4	1503.77368	96.4015
2	8.675	MM	0.3955	840.08264	35.40022	3.5985

Totals : 2.33452e4 1539.17390

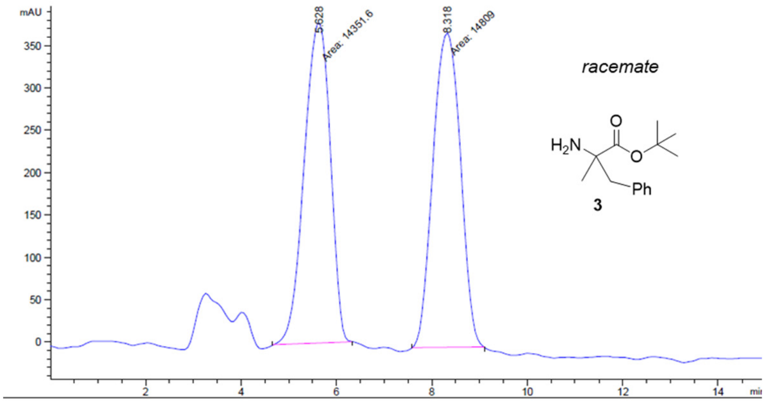

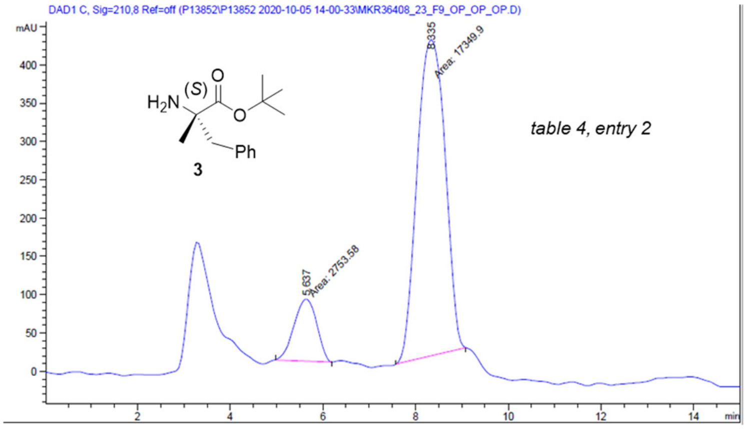

Peak #	RetTime [min]	Type	Width [min]	Area [mAU*s]	Height [mAU]	Area %
1	5.637	MM	0.5645	2753.57593	81.29742	13.6970
2	8.335	MM	0.6994	1.73499e4	413.47247	86.3030

Totals : 2.01035e4 494.76990

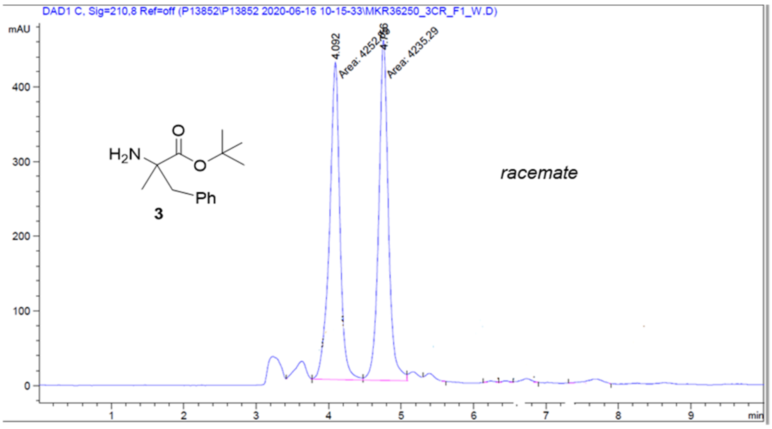
DAD1 C, Sig=210,8 Ref=off (P13852\P13852 2020-06-16 10-15-33\MKR36250_3CR_F1_W.D)
mAU
4.092
Area: 4252.46
Area: 4235.29
H2N
O
O
Ph
3
racemate
min

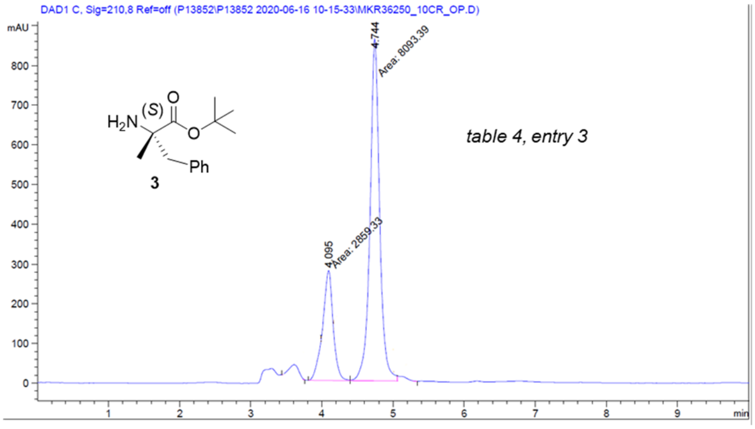
DAD1 C, Sig=210,8 Ref=off (P13852\P13852 2020-06-16 10-15-33\MKR36250_10CR_OP.D)
mAU
4.744
Area: 8093.39
4.095
Area: 2859.33
(S)
H2N
O
O
Ph
3
table 4, entry 3
min
